# The Molecular Genetics of von Willebrand Disease

**DOI:** 10.5505/tjh.2012.39205

**Published:** 2012-12-05

**Authors:** Ergül Berber

**Affiliations:** 1 İstanbul Arel University, Department of Molecular Biology and Genetics, İstanbul, Turkey

**Keywords:** Von Willebrand factor, von Willebrand disease, Von Willebrand factor biosynthesis

## Abstract

Quantitative and/or qualitative deficiency of von Willebrand factor (vWF) is associated with the most common inherited bleeding disease von Willebrand disease (vWD). vWD is a complex disease with clinical and genetic heterogeneity. Incomplete penetrance and variable expression due to genetic and environmental factors contribute to its complexity. vWD also has a complex molecular pathogenesis. Some vWF gene mutations are associated with the affected vWF biosynthesis and multimerization, whereas others are associated with increased clearance and functional impairment. Moreover, in addition to a particular mutation, type O blood may result in the more severe phenotype. The present review aimed to provide a summary of the current literature on the molecular genetics of vWD.

**Conflict of interest:**None declared.

## Abstract (tr)

Von Willebrand Faktöründe (vWF) görülen kantitatif azalma veya kalitatif bozukluklar bir kanama diyatezi olan ve oldukça sık rastlanan von Willebrand Hastalığının (VWH) oluşumuna neden olur. VWH klinik ve genetik heterojenite gösteren karmaşık bir hastalıktır. Genetik ve çevresel nedenlere ikincil eksik penetrans ve VWF düzeyinde olan değişiklikler olması VWH’nın karmaşık bir yapı göstermesine neden olan faktörlerdir. VWF genindeki mutasyonlardan bazıları biyosentezi ve multimerizasyonu etkilerken, diğer mutasyonlar vWF’nin dolaşımdan daha erken uzaklaştırılmasına neden olarak fonksiyonunu etkiler. Belirli bir mutasyon ile birlikte kan grubunun da O olması fenotipin daha ağır olmasına neden olabilir. Bu derlemenin amacı vWH’nın moleküler genetiği ile ilgili güncel yayınları inceleyerek onların bir özetini yapmaktır.

## INTRODUCTION

Von Willebrand disease (vWD) was first describedby Erik von Willebrand in a large Aland Islands (Swedish-speaking region of Finland) family in 1924 and wasreported to be the most common inherited bleedingdisorder in 1926 [1,2]. Its prevalence is estimated to be0.6%-1.3%; however, 1 in 10,000 patients have significantbleeding [3,4,5]. The characteristic clinical symptoms of vWD affect both males and females, and include mucosalbleeding (including epistaxis), menorrhagia, and prolongedbleeding following trauma or surgery. Severelyaffected patients may also bleed into soft tissues and joints[6].

vWD is associated with qualitative and quantitative deficiencyof von Willebrand factor (vWF). Clinical diagnosisof vWD is based on von Willebrand antigen (vWF:Ag), factor (F) VIII clotting activity (FVIII:C), and von Willebrandristocetin cofactor activity (vWF:RCo). Accordingto The International Society of Thrombosis and Haemostasis(ISTH), vWD is classified as type 1, type 2, and type3 (Table 1). Type 1 and type 3 vWD are characterizedby partial and complete deficiency of vWF, respectively.Functional deficiency of vWF is characteristic of type 2vWD, which is further classified as 2A, 2B, and 2M basedon defective interaction with platelets, and as 2N based ondefective binding to the FVIII molecule. Although ISTHclassification of 1994 indicated that vWD is a disease associatedwith vWF gene mutations, the 2006 revised ISTHclassification states that, “additional genes might influencethe biosynthesis and stability of plasma vWF” [7]. 

**von Willebrand Factor**

**Figure 1:**
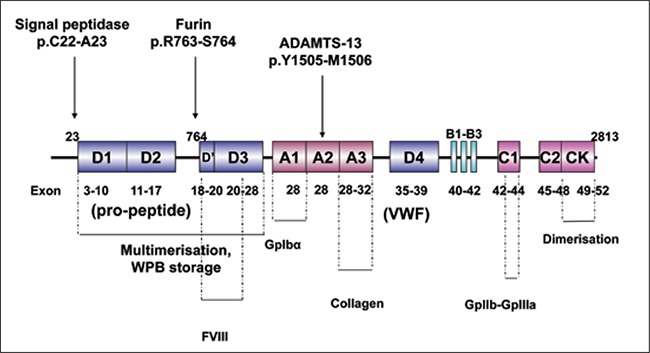
Structure andfunctional domains ofpre-pro-VWF.

vWF is a multimeric plasma glycoprotein essential forprimary hemostasis that mediates platelet plug formationvia adhesion at the site of injury. In addition, vWF protectsFVIII in plasma from proteolytic degradation by noncovalentlybinding to it and transports FVIII to the site ofcoagulation [8]. vWF is produced in endothelial cells andmegakaryocytes as a pre-pro-polypeptide (pre-pro-vWF)that is 2813 amino acids long. Pre-pro-vWF is composedof a signal sequence that is 22 amino acids long, a pro-peptidesequence 741 amino acids long, and a vWF monomersequence 2050 amino acids long (Figure 1) [9,10,11].

During vWF biosynthesis the signal sequence is cleavedoff by a signal peptidase after it is directed to the endoplasmicreticulum. Pro-vWF monomer undergoes dimerizationat the CK domain via disulfide bonding betweencysteine residues. In addition to dimerization, vWF isglycosylated in the endoplasmic reticulum. The dimersare then transported to the Golgi apparatus where theyundergo amino terminal multimerization, sulfation, andfurther glycosylation, and change carbohydrates into highmannose oligosaccharides. Finally, propeptide is cleavedfrom the vWF multimers by furin (PACE [paired basicamino acid cleaving enzyme]) and remains non-covalentlyassociated with the vWF multimers; 95% of vWF multimersare secreted constitutively and 5% of plasma vWFmultimers are stored in the Weibel-Palade bodies (WPBs)of endothelial cells and are secreted in a regulated mannerupon stimulation by thrombin, fibrin, and histidine[4,9,11,12,13,14,1^5^,^16^]. 

Research has shown that the pro-peptide sequence actsas a chaperone to direct vWF multimers to WPBs [17].Glycosylation of vWF is an important post-translationalmodification that protects vWF from proteolytic destruction,affects plasma clearance, and maintains vWF’s multimericstructure and interaction with platelets and collagen[18,19,20]. vWF multimers range in size from a dimer (~500kDa) to ultra large multimers (>10 x 106 kDa). vWF multimersreleased from WPBs are ultra large (UL-vWF) and arethe most effective for maintaining hemostasis at the site ofinjury. Pro-peptide’s Cys-X-X-Cys sequence, similar to theactive site of protein disulfide isomerases, is thought to beimportant for vWF multimer formation [20,21].

UL-vWF multimers are proteolytically cleaved byvWF cleaving protease (ADAMTS-13) to physiologicallyactive plasma vWF multimer size within the A2 domain(Y1605-M1606) [22, 23]. The A2 domain serves as ashear sensor and its unfolding is necessary for proteolyticcleavage to expose the cleavage site [24]. In addition tothe importance of the cleavage site in the A2 domain forADAMTS-13 proteolysis, the residues C1669 and C1670that form a disulfide bond are important for A2 domainfolding [25]. Furthermore, studies have shown that polymorphismsin the A1 and A2 domains affect the efficiencyof ADAMTS-13 cleavage [26].

The vWF gene spans a 178-kb genomic region with 52exons; exon 28 is 1379 bp long and is the largest exon.Exon 28 encodes the domains involved in FVIII binding(D3), platelet binding (A1), collagen binding (A3), andADAMTS-13 cleavage (A2). There is a highly homologouspseudo-gene containing the vWF gene region from exon23 to 34 on chromosome 22 (22q11.2) [27,28].

The half-life of vWF in circulation is 12-20 h. The vWFplasma level ranges between 50 IU/dL and 200 IU/dL inthe general population [29]. Twin studies reported thatgenetic factors are responsible for 60% of the variation in the vWF plasma level, which is also affected by a varietyof other factors, including age, blood type, stress, thyroidhormone, pregnancy, single-nucleotide polymorphisms(SNPs) in the vWF gene—including the promoter regionand other genetic loci [30,31,32,33]. Recent studies reportedthat macrophages are involved in the removal of vWF/FVIII complex in the liver and spleen, and that D’-D3domains are implicated in the clearance, although the precisemechanism of vWF clearance remains unknown [34-35]. Blood type is a major genetic determinant of the vWFplasma level [30]; it was reported that individuals withtype O blood have 25% less vWF due to increased susceptibilityto cleavage by ADAMTS-13 [36].

**Type 3 vWD**

**Figure 2:**
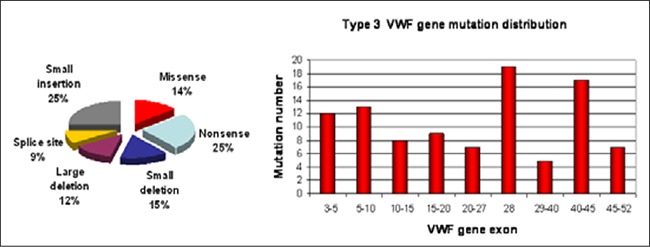
Types anddistribution of the VWF genemutations identified in Type3 VWD patients [39,41]

Type 3 vWD is characterized by the virtual absenceof plasma vWF and a consequent decrease in the FVIIIlevel to 10%. The frequency of type 3 vWD is between 0.5and 5.3/1,000,000 individuals [3^7^,^38^]. Type 3 vWF genemutations are recessive and such patients are homozygousor compound heterozygous for the vWF gene mutationthat creates a null allele [39,40]. Nonsense mutations arethe most common of the wide range of type 3 mutationsscattered throughout the gene, most of which are in exon28 (Figure 2). The most common nonsense mutation isR1659X in exon 28 [41]. Nonsense-mediated decay of theallele-specific mRNA is thought to be the molecular mechanismof the nonsense mutations [42].

Missense mutations are the second most commonmutations, some of which result in the replacement of cysteineresidues that might cause multimerization and secretiondefects [37,43,44]. Deletions resulting from recombinationevents include a single exon deletion, multipleexon deletion, and whole gene deletion. The most commondeletion in the vWF mutation database is c.2435delCin exon 18. Deletion of exons 4 and 5 is reported to bea recurrent deletion in the UK, and is associated with a common haplotype and founder effect [45]. Although it isa rare complication, development of alloantibodies againstvWF is observed in association with large deletions andwhole gene deletion (Table 2) [39,46,47]. Alu (shortstretch of DNA)-mediated recombination, impaired secretion,and multimerization are the causative mechanismsfor some of the deletions. In addition, gene conversionevents between the vWF pseudogene and the vWF genethat result in multiple substitutions and a stop codon inthe vWF gene are reported to be a common pathogenicmechanism in type 3 vWD patients [48,49].

**Type 2 vWD**

There is a functional deficiency of vWF in patients withtype 2 vWD, which is further classified as 2A, 2B, and 2Mbased on defective interaction with platelets, and as 2Nbased on defective binding to the FVIII molecule. 

**Type 2A vWD**

**Figure 3:**
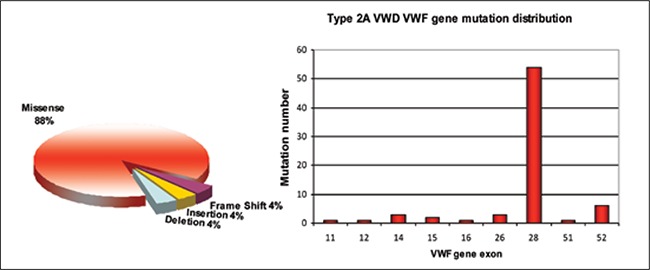
Types anddistribution of the VWF genemutations identified in Type2A VWD patients [39,41]

Type 2A vWD is characterized by defective plateletbinding due to the absence of high molecular weight vWF (HMW-vWF) multimers in both plasma and platelets.Type 2A vWD patients have a low vWF:RCo to vWF:Agratio (<0.6) [37]. Type 2A mutations include missense,deletion, insertion, and frameshift mutations; 73% ofthese mutations are located within exon 28 (Figure 3) and90% of these mutations are missense mutations that areeither recessive or dominant [40]. D2 domain mutationsare recessive and prevent multimer formation; patientsare either homozygous or compound heterozygous with anull allele. Mutations in the D3, A2, A1, and CK domainsare dominant mutations. D3 and CK mutations inhibitmultimerization and dimerization, respectively [50,51].A2 and A1 mutations result in an increase in susceptibilityto ADAMTS-13 proteolysis, defective biosynthesis, orintracellular retention [52,53]. Type 2A vWD mutationsthat cause defective biosynthesis, intracellular storage, andintracellular retention (e.g. N528S) are group I mutations[54,55,56]; other mutations that increase ADAMTS-13 sensitivityare group II mutations (e.g. L1505R) [57]. GroupI mutations result in a more severe phenotype than dogroup II mutations and patients respond better to DDAVPtreatment [58].

**Type 2B vWD**

Spontaneous and increased binding of vWF to GpIbαreceptors on platelets due to dominant gain-of-function A1domain mutations and the absence of HMW-vWF multimersin plasma are characteristic of type 2B vWD. Dueto spontaneous platelet binding HMW-vWF multimers inplasma are proteolyzed by ADAMTS-13 [7,59]. Patientshave a low VWF:RCo to VWF:Ag ratio (<0.6) and anincrease in ristocetin-induced platelet aggregation (RIPA)at low dose ristocetin [37,60]. Thrombocytopenia is alsoobserved in some type 2B vWD patients under stress conditions,such as pregnancy or infection, and after DDAVPuse. Patients may have giant platelets and also suffer from homospontaneousplatelet agglutination [61,62,63,64,65]. Clinical manifestationof type 2B vWD varies and patients with thrombocytopeniahave the most severe phenotype [66]. Furthermore,mouse models of type 2B mutations R2306Qand V1316M show that both mutations and ADAMTS-13determine the phenotype [59].

**Figure 4:**
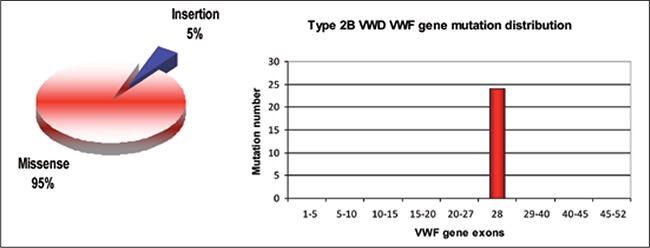
Types anddistribution of the VWF genemutations identified in Type2B VWD patients [39,41]

There are >50 type 2B mutation submissions in thevWF mutation database. Type 2B mutations are highlypenetrant and are detected only between codons 1266 and1461—the region of exon 28 of the vWF gene encodingA1 domain; 96% of these mutations are missense mutations,most of which are observed at mutation hotspotarginine codons at positions 1306 (R1306W/Q/L), 1308(R1308C/P), and 1341 (R1341Q/P/L) (Figure 4) [41]. Arecent type 2B genotype-phenotype study reported thatV1316M mutation is associated with the most severebleeding score, as compared to other type 2B mutations[66,67]. Moreover, some type 2B mutations are associatedwith dynamic changes in the vWF level in associationwith the platelet count. For example, it was reportedthat a patient with R1306W mutation had normalizedhigh molecular weight multimers (HMWM), but severethrombocytopenia and a decrease in HMWM after correctionof the platelet count [68]. In contrast to classical type2B mutations, P1266Q/L (New York/Malmö) and R1308Lmutations do not affect multimer size and do not causethrombocytopenia [66]. 

**Type 2M vWD**

**Figure 5:**
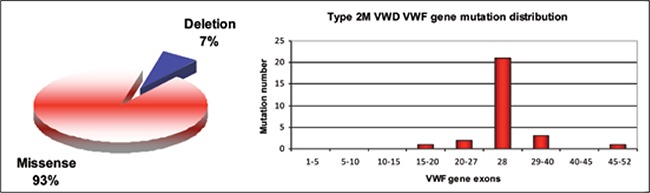
Types anddistribution of the VWF genemutations identified in Type2M VWD patients [39,41]

Type 2M vWD is characterized by a defect in vWFplateletbinding due to dysfunctional HMW-vWF causedby vWF gene mutations, despite a quantitatively normalvWF multimeric structure. Type 2M mutations are dominantloss-of-function mutations predominantly locatedwithin the platelet GP1b binding A1 domain [7,37]; 93%of these mutations are missense mutations and the remainderare small in-frame deletions [41]. Type 2M mutationsare fully penetrant and 75% occur in exon 28 of the vWFgene (Figure 5). A Canadian cohort study reported that avWF:RCo to vWF:Ag ratio <0.4 in type 2M vWD patientswas strongly associated with A1 domain mutations [69].There are also a small number of mutations in the A3domain (S1731T, W1745C, and S1738A) that affect collagenattachment and cause mild bleeding. Despite thefact that type 2M vWD patients respond poorly to DDAVPtreatment, patients with A3 domain mutations respondwell to DDAVP [70,71].

**Type 2N vWD**

Type 2N vWD is characterized by markedly reducedor lack of vWF affinity FVIII binding. Recessive mutationsin the vWF-FVIII binding domain result in the lackof FVIII binding and a disproportionate decrease in theFVIII:C level to between 0.05 and 0.30 IU/mL. The type2N vWD phenotype is observed in patients that are homozygous for the same FVIII binding mutation, compoundheterozygous for 2 different FVIII binding mutations, orcompound heterozygous for a FVIII binding mutation anda vWF null allele [37,72,73,74]. vWF binds to FVIII throughits D’ domain and part of the D3 domain between residuesSer764 and Arg1035 encoded by exons 18-23 inthe vWF gene [75]; however, mutations beyond the FVIIIbinding regions (from exon 23 to 27) are also associatedwith decreased FVIII binding (e.g., Q1053H and C1060R)[76,77].

**Figure 6:**
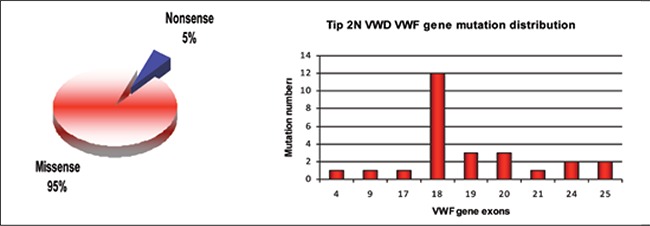
Types anddistribution of the VWF genemutations identified in Type2N VWD patients [39,41]

In addition to FVIII binding impairment, type 2Nmutations might also cause secretion and multimerizationdefects, especially cysteine mutations (C788R/Y, Y795C,and C804F) [78]. Moreover, the 2 pro-peptide mutationsR760C and R763G sterically inhibit FVIII bindingby preventing furin cleavage of pro-peptide, resulting inthe formation of UL-vWF multimers [37,74]. Type 2Nmutations occur primarily between exon 18 and 20 [41];95% of the mutations are missense mutations (Figure 6).R816W and R854Q are the most common type 2N mutations.Type 2N mutations are highly penetrant and thelevel of FVIII in type 2N vWD patients is associated withthe specific mutation. For example, R816W mutationleads to a severe decrease in the FVIII level (<10 IU/mL)and patients do not respond to DDAVP, whereas R854Q mutation is associated with a less severe phenotype and aFVIII level of 20 IU/mL, and such patients do respond toDDAVP [78].

**Type 1 vWD**

Type 1 vWD is characterized by partial quantitativedeficiency of functionally normal vWF. The level of vWFis reduced to between 5 and 50 IU/dL, without significantabnormalities in multimer structure [5]. It is generallyinherited as autosomal dominant; however, its clinicaldiagnosis is complicated due to incomplete penetranceand variable expression of the vWF gene [79]. In addition,compound heterozygosity for type 3 or type 2N mutationsinfluence the severity of the disease. Recent studiesperformed in the European Union, the UK, and Canadahave provided some data on the molecular pathology oftype 1 vWD and established that there is a genotype-phenotypecorrelation [80,81,82]; the vWF gene was analyzed in~300 type 1 vWD patients in the 3 studies, which demonstratedthat both allelic and locus heterogeneity should beconsidered to play a role in the molecular pathogenesis oftype 1 vWD.

**Figure 7:**
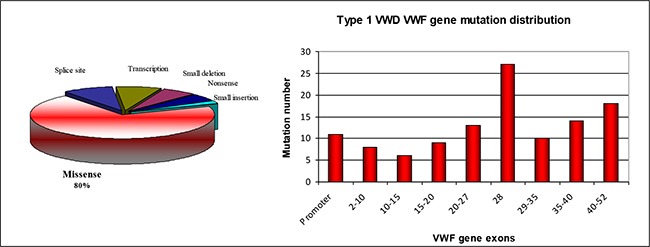
Types anddistribution of the VWF genemutations identified in Type1 VWD patients [39,41]

Many candidate mutations, including promoter, splicesite, nonsense, missense, and small insertions, as wellas deletions have been identified; 80% of the mutations pathoaremissense mutations and mutations primarily occur inexon 28 (Figure 7). In addition, some patients have morethan vWF gene mutations. One of the major findings ofthese studies is that type 1 vWD is not always related tovWF gene mutations. Candidate mutations were identifiedonly in 65% of patients and are more likely in patientswith vWF:Ag <30 IU/dL. Moreover, mutation penetranceincreases as the vWF plasma level decreases [3^7^,^81^]. TypeO blood type is associated with type 1 vWD in patientslacking any identified vWF gene mutation [39,81]. Studiesthat examined expression of the candidate mutationsnoted 2 primary pathogenetic mechanisms in type1 vWD. The first mechanism is intracellular retention ofthe mutant vWF gene. Some vWF gene mutations, such asC1149R, were shown to dominantly impair vWF secretion[83,84]. Type 1 mutations that cause a loss or increase incysteine residue (C2257S or G2441C) affect biosynthesisby causing significant intracellular retention and loss ofmultimeric structure. In contrast to this, D4 domain mutationL2207P caused similar significant intracellular retentionand multimer loss to the mutations that involve Cysresidue [85].

The second pathogenic mechanism is accelerated vWFclearance, which causes a very brief response to DDAVP inpatients (<4 h), as compared to healthy individuals (6-9h), and an increase in the vWF pro-peptide (vWFpp) tovWF:Ag ratio. Due to the clearance this type of vWD is alsoknown as type 1C vWD. The mutations associated withaccelerated vWF clearance are R1205H (vWD Vicenza),C1130G/F/R, W1144G, I1416N, and S1279F [5,86,87,88]. 

Research has shown that ABO blood types also influencevWF clearance and the severity of the phenotype invWF gene mutation carriers. For example, Y1584C mutationwas the most common type 1 vWD mutation in 3type 1 studies with incomplete penetrance. Although thismutation causes intracellular retention, all symptomaticY1584C carriers also had type O blood in the Canadianand UK type 1 vWD studies, and patients in the UKstudy had an elevated vWFpp to vWF:Ag ratio [89,90,91,92].Similarly, C2362F carriers with type O blood had a moresevere phenotype [93].

There are also some common type 1 vWD mutationsfor which the molecular pathogenesis has yet to be discerned.For example, R924Q is a recurrent mutationassociated with a founder haplotype and marks a splicingdefect that created a null allele in a Canadian patient thatwas a compound heterozygous for R816W type 2N mutation;however, other studies reported that R924Q variationis a polymorphism [94,95]. 

**Genetic testing in vWD**

Genetic testing of patients with inherited diseases hasan important role in expanding our understanding of themolecular pathology of such diseases, and in decreasingdisease-related morbidity and mortality. For some inheritedcomplex disorders, including maturity onset diabetesof the young (MODY), genetic testing is important for differentiatingdisease subtypes and determining the optimaltreatment method. Moreover, prenatal genetic diagnosis isextremely important for decreasing the frequency of inheriteddiseases as well as limiting the psychological and economicconsequences for patients and their families. 

vWD is a complex inherited bleeding disorder withclinical and genetic heterogeneity. Incomplete penetranceand variable expression are the major roadblocks to clinicaldiagnosis. Clinical diagnosis of vWD is based on phenotypicdata; however, high variation in assays or lowerdetection limit, particularly vWF:RCo, and unavailabilityof certain tests like vWF:FVIIIB (vWF:FVIII bondingassay) or multimer analysis would also lead to misdiagnosisor inefficient diagnosis of vWD. Genetic testing ofpatients with vWD is based on vWF gene analysis. Thevalue of genetic testing in vWD depends on the subtype;it is useful for the differential diagnosis and determiningthe proper treatment in patients with type 2 vWD. Genetictesting could be helpful in differentiating type 2N vWDfrom hemophilia A, which is possible by analyzing theexons encoding the FVIII binding region (exons 17-25).Genetic testing could also be useful for differentiating type2B vWD from platelet-type-vWD, which is based on analyzingjust exon 28 in the vWF gene. In addition, genetictesting is important for the correct diagnosis of type 2A andtype 2M vWD if multimer analysis cannot be performed.Genetic diagnosis of type 2A and type 2M vWD could alsobenefit the treatment of vWD, as type 2A patients respondto DDAVP, whereas type 2M patients do not. Clinicaldiagnosis of type 3 vWD is easily made based on phenotypictesting, as vWF is completely absent in the plasma.Nevertheless, genetic testing of type 3 vWD patients couldbe used for genetic counseling, prenatal diagnosis, andpredicting inhibitor formation; however, the whole genemust be analyzed because mutations are scattered alongthe vWF gene. 

On the other hand, because correctly diagnosing type 1vWD is clinically problematic and due to partial deficiencyof vWF, molecular diagnosis is also problematic becauseof the complexity and mutational heterogeneity of thevWF gene. Many candidate mutations have been identifiedin type 1 vWD patients; in vitro expression studies areimportant for determining whether or not they are pathogenic variations. Hence, expression analysis of some candidatemutations showed they are just neutral polymorphisms.For some sequence variations, such as R924Q, thepresence of a specific haplotype might be responsible forthe disease phenotype. Moreover, it is likely that ≥35%of type 1 vWD patients do not have any vWF gene mutation.Finally, the presence of incomplete penetrance andthe complex pathogenesis of vWD are major limitations tomaking a genotype-phenotype association in type 1 vWDpatients. Consequently, although the use of genetic testingin type 1 vWD is of limited use, it could be used inpatients with vWF:Ag <30% and in those with mutationsthat affect vWF clearance, such as R1205H mutation, fordifferentiating type 1 vWD from type 2 vWD [96,97,98].

## CONCLUSION

ConclusionvWD is an inherited bleeding disorder with a complexmolecular pathology. Although numerous studies invarious geographic regions have considerably advancedour understanding of the molecular mechanism of vWD,cases of vWD not associated with vWF gene defects arestill observed. Complete understanding of the molecularpathogenesis of vWD requires additional in vitro expressionstudies that observe the effects of the candidatevWF gene mutations. In addition, use of whole genomeor exome (part of genome formed by exons) sequencing(novel technologies) might identify other genetic determinantsof vWD and help to complete our understanding ofvWD by demonstrating the genotype-phenotype relationship.

**Conflict of Interest Statement**

The authors of this paper have no conflicts of interest,including specific financial interests, relationships, and/or affiliations relevant to the subject matter or materialsincluded.

## Figures and Tables

**Table 1:**
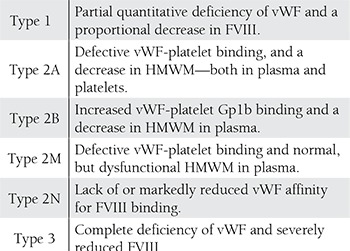
vWD types.

**Table 2:**
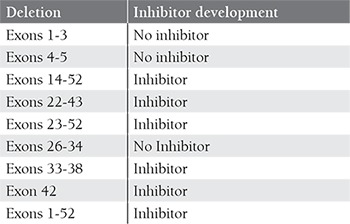
vWF gene deletions and inhibitor development.
